# Physiological Discoveries

**Published:** 1837-07

**Authors:** T. J. Todd


### PHYSIOLOGICAL DISCOVERIES. BY T. J. TODD, M.D.

I. *Nutrition and Secretion.* Dr. Todd, of Brighton, in pursuing a course of physiological researches, has been led to the conclusion that the process of nutrition as well as of secretion is performed by capillaries in a state of *stasis.* The capillaries on the surface of a granulating ulcer are in this state. This form of stasis of the blood in the capillaries, which, in contradistinction to other forms, may be termed *dynamic* stasis, is not a passive but an active state of these vessels, entirely independent of the heart, but dependent upon their own innervation, or that of the organs to which they belong. Dr. Todd has been able to determine the whole process of the formation of new vessels, differing in some respects from that of previous observers; and he has ascertained by direct experiment the conversion of coagulable lymph into pus, without the intervention of the function of any description of vessels.

II. *Artificial Digestion.* Dr. Todd, with the assistance of Mr. Schweitzer, of the German Spa, Brighton, has been performing experiments with the artificial *digestive fluid,* in imitation of those of Schwann, of Berlin, recorded in our present number, and has arrived at some new and interesting results not attained by Dr. Schwann. The digestive fluids with which Dr. Todd operated were prepared from the stomachs of the ox, the horse, the dog, and the cat. Some, also, prepared from the upper portion of the small intestines, was found not less powerful. The presence of the acid is essentially necessary in the preparation; when Mr. Schweitzer endeavoured to procure the digestive fluid with distilled water alone, or when he treated the mucous membrane in the same way by a weak alkaline solution, a rapid putrefaction stopped all further proceeding. Various vegetable and animal substances were submitted to the action of these digestive fluids, at the ordinary temperature of the atmosphere, and the constant result in all the instances has been that all these substances have been resolved into their elementary organic globules. There has been no exception to this, so far as the experiments have extended, and these include, amongst vegetable substances, the artificial digestion of boiled cauliflower, of bread, and of vermicelli, not dressed; and, amongst animal substances, the white of egg boiled, the coagulum of blood, butter, fat, the muscular fibre of mutton, and of fish boiled and raw, filaments of the sciatic nerve raw, and scrapings of bone. The products of these artificial digestions, especially of the vegetable substances, compared with chyme taken from a dog which had been feeding upon ground oats, were very much alike, except that the watery part had been removed from the chyme. It is therefore reasonable to conclude, that the process of artificial digestion is essentially the same with the process of natural digestion in its first stage, that alimentary substances are not reduced to their chemical, but resolved into their organic elements; so that the globules, observed by physiologists in the stomach, are not formed by the process of digestion, but are the globules existing in the alimentary substances, developed or disengaged by that process.

At another time we hope to be able to lay before our readers some account of the researches which have led to these important discoveries.

*British Annals of Medicine.* No. 16 and 23.

